# Experimental investigation of the information entropic Bell inequality

**DOI:** 10.1038/srep23758

**Published:** 2016-04-01

**Authors:** Lian-Zhen Cao, Jia-Qiang Zhao, Xia Liu, Yang Yang, Ying-De Li, Xiao-Qin Wang, Zeng-Bing Chen, Huai-Xin Lu

**Affiliations:** 1 Department of Physics and Optoelectronic Engineering, Weifang University, Weifang, Shandong 261061, China; 2 Hefei National Laboratory for Physical Sciences at Microscale and Department of Modern Physics, University of Science and Technology of China, Hefei, Anhui 230026, People’s Republic of China

## Abstract

Inequalities of information entropic play a fundamental role in information theory and have been employed effectively in finding bounds on optimal rates of various information-processing tasks. In this paper, we perform the first experimental demonstration of the information-theoretic spin-1/2 inequality using the high-fidelity entangled state. Furthermore, we study the evolution of information difference of entropy when photons passing through different noisy channels and give the experimental rules of the information difference degradation. Our work provides an new essential tool for quantum information processing and measurement, and offers new insights into the dynamics of quantum correlation in open systems.

Braunstein and Caves first conceptually proposed a two-party N-setting information entropic Bell inequalities (BC chained Bell inequalities). It was shown that local realism imposes nontrivial conditions already on the level of the Shannon entropies. The Shannon entropies carried by the measurements on two distant systems must satisfy certain inequalities^1^,^2^. The information entropic Bell inequalities have some interesting applications in situations where the CHSH Bell inequalities are inadequate. For instance, the information entropic Bell inequalities can readily be applied to quantum systems of arbitrary local dimension and general measurement operators. Since the inequalities are independent on the number of outcomes of the measured observables, they can reduce the number of trials needed to rule out local realism in experiments^3^.

Especially, with the rapid development of quantum information theory and applications, information entropic inequalities are used as a fundamental tool in the context of quantum information processing and measurement^4^. For instance, the use of information entropic Bell inequalities in quantum computation to solve “database” search problem^5^ and marginal problems^6^, to test quantum contextuality^7^, to demonstrate the entanglement swapping experiment^8^, and to study the general correlation and causal model scenarios^9^,^10^, to mention a few. Moreover, the information entropic Bell inequalities with higher values of N (the number of measurable quantities) can improve the security of quantum key distribution protocols^11^, and have been also used to investigate nonlocal theories^12^,^13^. Furthermore, analogous to the entropic Bell inequalities derived by Braunstein and Caves, entropic properties of quantum states have been widely studied^14^ in the framework of q-deformed entropic functions like the Reniy entropy^15^ and the Tsallis entropy^16^. All the mentioned above information and entropic schemes for quantum systems open new prospectives for implementation of quantum technologies, e.g., realization of quantum algorithms, quantum memory devices, and many-level quantum simulation^17^. However, experimental demonstration and verification of the entropic inequalities are very difficult due to the stringent requirement of system states^13^,^18^. To our knowledge, the experimental report on information entropy inequalities is very few.

An unavoidable coupling between a real quantum system and its environment can cause decoherence, leading to the destruction of quantum correlation among subsystems simultaneously. This in turn can hinder the success of quantum information protocols. Expecially, how to characterize and extract the information of quantum noisy channels is essential not only for designing optimal dynamical protection from decoherence caused by a given environment of quantum information processing, quantum-state transfer, Hamiltonian engineering, and quantum state storage, but also for playing a fundamental role on understanding physical processes^19^,^20^,^21^,^22^,^23^,^24^. Therefore, the characterization of quantum correlation evolution under the influence of decohering processes is required for any future realization of these quantum information applications and some works have been reported in several papers^25^,^26^,^27^. However, information theoretic formulations of the quantum correlation in the presence of decoherence and noise were not addressed by now.

In this paper, for the first time we experimentally demonstrate the information-theoretic spin-1/2 Bell inequality. Moreover, we study the information difference degradation in the bit-flip and phase-shift noises environment and give the experimental rules of the information difference evolution. The results show that the correlation between space-like separated entangled quantum system can be quantified using the quantum information entropy.

## Results

### Theoretical model

Braunstein and Caves proposed an N-setting generalization of the CHSH Bell inequality. Consider two counter-propagating spin-s particles, A and B, and N = 2Q measurable quantities-*A*_1_, ···, *A*_*Q*_ associated with A and *B*_1_, ···, *B*_*Q*_ associated with B-interleaved in a sequence *A*_1_, *B*_*Q*_, *A*_2_, *B*_*Q*−1_, ···, *A*_*Q*−1_, *B*_2_, *A*_*Q*_, *B*_1_. Objectivity and locality justify a joint probability for the N quantities. Then the information Bell inequality can be obtained


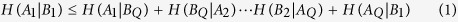


The *H*(*A*|*B*) is the information carried by A, given the value of B.

To investigate violation by quantum mechanics, we must return to the two spin-s particles considered above. The N measurable quantities, 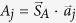 and 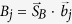 for *j* = 1, 2, ···, *Q* are spin components specified by unit vectors 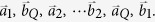 If these vectors are coplanar and successive vectors in the list are separated by angle 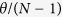, then the chained information Bell inequality is violated when the information difference


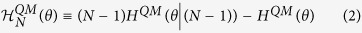


is negative. The quantum mechanical information *H*^*QM*^(*A*|*B*) = *H*^*QM*^(*B*|*A*) ≡ *H*^*QM*^(*θ*) takes the form 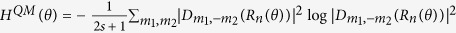, where 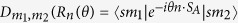 is rotation matrix and this can be calculated from the relation 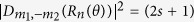

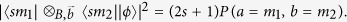


The above quantum statistics can be derived from the state of zero total spin: 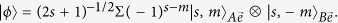 A spin-1/2 particle has two states (|−1/2〉) and (|1/2〉), and can be given by


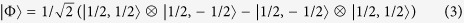


This state can be experimental realized using the polarization light field produced by pulsed type-II parametric down-conversion. If we only consider the first order term of parametric down-conversion, the quantum state 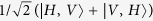 is prepared, where H and V are horizontal and vertical polarization of photon, respectively. If we encode the |1/2, 1/2〉 and |1/2, −1/2〉 with the photon’s horizontal (H) and vertical (V) polarization, the quantum state is just the state (3).

### Experiment model

**Figure 1:**
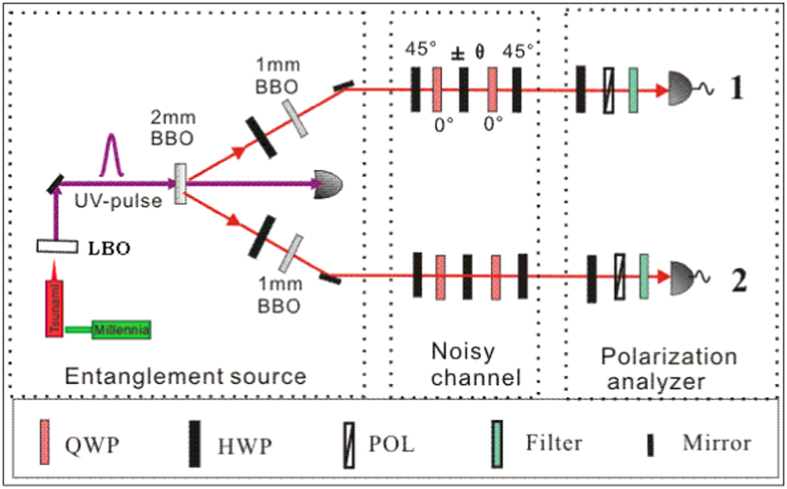
Schematic drawing of the experimental setup for the generation and detection of high fidelity two qubit entangled states and for engineered collective and non-collective noises.

The experimental set-up is shown in Fig. 1. Ultraviolet laser pulses with a central wavelength of 390 nm, pulse duration of 100 fs, and a repetition rate of 80 MHz pass through one *β*-barium borate (BBO) crystal with a thickness of 2 mm to produce two entangled photon pairs. The photons pass through a pair of birefringent compensators consisting of a half-wave plate (HWP) and a 1-mm BBO crystal to compensate the walk-off between horizontal and vertical polarization, and are prepared in the quantum state 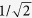
(|*H*, *V*〉 + |*V*, *H*〉). Output photons are guided to noise engineered set-up, and then well coupled into two single-mode fibers and finally detected by two single-photon counting modules. In order to get the highest fidelity of our output states, the average pumping power of the laser is selected as 40 mW. It must be noted that the experimental verification of the entropic inequalities is very difficult due to the stringent fidelity requirements of system states^1^,^5^,^6^,^7^,^8^,^9^,^10^. Thus, we take many methods to improve the fidelity of entangled state and obtain the fidelity for the two particle entangled state to be 0.997 ± 0.001, thus, prove the presence of genuine two particle entanglement^28^.

We experimentally simulate the collective and non-collective rotation noise (bit-flip and phase-shift noise) models. For the collective noise, all the qubits rotate along the Y-axis with a random angle. For simplicity, we chose the angle to be ±*θ* with an equal probability. Note that, for a single qubit, this is nothing but a bit flip noise with probability 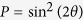. The non-collective bit-flip noise is achieved by setting the angle of the HWP in each channel to be ±*θ* independently. Experimentally, the noise channels were engineered by setting the angles of half-wave plates to be ±*θ* with an equal probability, and each HWP was sandwiched by two quarter-wave plates (QWPs) at 0° with respect to the vertical direction. Furthermore phase-shift noise is also simulated by adding two HWPs each setting at 45° before and after the wave-plates.

## Discussion

**Figure 2:**
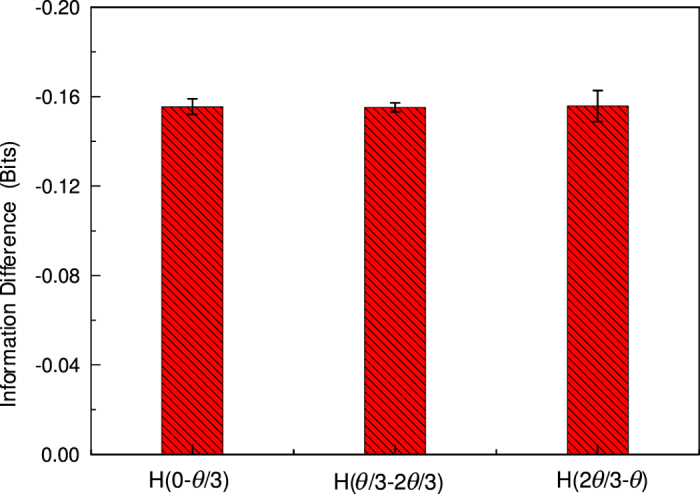
The quantum-mechanical information equivalent relation when the rotation angle from 0 to *θ*/3, *θ*/3 to 2*θ*/3 and 2*θ*/3 to *θ*. The angle *θ* is 52.31° corresponding to the location of maximum theoretic information deficit. Error bars represent statistical errors.

To verify the information entropic theoretic prediction, we first experimentally give the equivalent relation among the *H*^*QM*^(0, *θ*/3), *H*^*QM*^(*θ*/3, 2*θ*/3), and *H*^*QM*^(2*θ*/3, *θ*) according to the rotational invariance and the symmetry of the experimental system. This step is significantly to decrease the amount of experimental measurement observables. In quantum mechanical description the two observables associated with each system would not commute and hence could not be determined simultaneously. Thus we have in mind a series of experimental runs, in each of which one measures only two quantities, one from each system. Since the two systems are widely separated, a measurement on one should not disturb the other. Based on the locality, the no-disturbance assumption means that the statistics of runs that measure a particular pair of quantities are given by the appropriate pair probability. The experimental results are shown in Fig. 2. It can be seen that the quantum-mechanical information *H*^*QM*^(0, *θ*/3), *H*^*QM*^(*θ*/3, 2*θ*/3), and *H*^*QM*^(2*θ*/3, *θ*) are equal within the error range.

**Figure 3:**
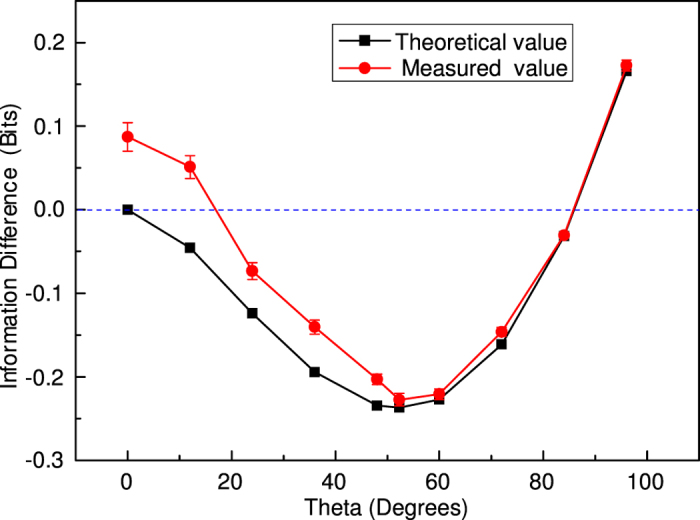
The information difference of theoretical calculated and measured results in bits vs angle in degrees for s = 1/2. The obviously difference between the theoretic and experimental value at the small-angle zone is caused by small angle effect. The data acquisition time is 60 seconds for each measuring point, collecting about 100 events.

The theoretic and experimental results of information difference for information Bell inequality in bits are shown in Fig. 3. The black solid line is the theoretic result whereas the red solid line represents experimental result. It can be seen that quantum conditional entropies can be negative for entangled systems, which leads to a violation of entropic Bell inequalities. The negative value means the deficit information carried by the two particles, relative to the requirement of local realism for this geometry. The maximum theoretic information deficit for spin-1/2 is −0.2369 bits at 52.31°. We obtained an experimental value of −0.227 ± 0.007. The experimental results agree well with the theoretical calculation. It should be point out that the difference between the theoretic and experimental value is bigger at the small-angle zone, which can be explained by the quantum Zeno paradox^1^. For any *θ*, when N is sufficiently large, the quantum mechanical information can be approximated by the small-angle behavior, but the N-1 measurements at the small angle *θ*/(*N* − 1) together yield vanishingly small information because of the tight correlation between the spins.

As mentioned above in introduction section, the investigation of the dynamics of quantum correlation for the quantum systems under the influence of decoherence has very important practical significance. Usually one has to deduce the time evolution of quantum correlation of the composite system from the time evolution of the quantum state under consideration. Much effort has been devoted to understanding the dynamics of entanglement^29^,^30^,^31^. Instead of deducing the evolution of entanglement from the time evolution of the state, Thomas Konrad *et al.*^32^ provided a direct relationship between the initial and final entanglement of an arbitrary bipartite state of two qubits with one qubit subject to incoherent dynamics. Here, a new measurement method based on an information difference is introduced to quantify the quantum correlation evolution between different parties in collective and non-collective bit-flip and phase-shift noises environment. Analogous to the method proposed by Thomas Konrad, our measurement method also did not consider time evolution, but only relies on the difference of quantum information carried by the entangled particles.

**Figure 4:**
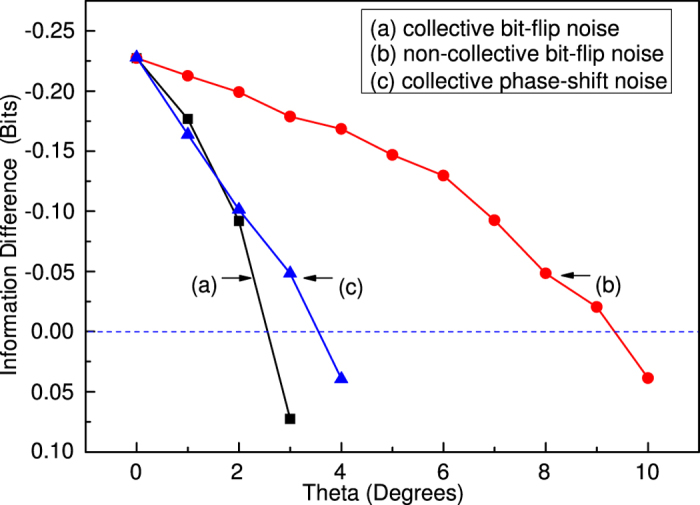
Evolution of the information difference under simulated noise. (**a,b**) the experimental information difference of S = 1/2 Bell inequality under the collective and non-collective bit-flip noise. (**c**) the experimental information difference of S = 1/2 Bell inequality under the collective phase-shift noise.

The experimental results are shown in Fig. 4. In Fig. 4(a,b) we give the change of information difference depending on the rotation angle of the HWP under the collective and non-collective bit-flip noises environment. The zero information difference value is the critical point to estimate the quantum nonlocality and quantum correlation still existing or not between the two bit entangled system. The general behavior shows that the quantum correlation decreases as the noise intensity increases. However, the decreasing rate depends on the type of the noise–correlated or non-correlated and noisy channels–bit-flip or phase-shift. For the non-collective bit-flip noise, the information difference is still negative when the rotation angle is 9.3°. While for the collective bit-flip noise, the upper bound of rotating angles to destroy the quantum correlation property is 2.5°. By comparing the experimental results, it is obvious that the quantum correlation decays smoothly to reach its minimum bounds for collective noise. Figure 4(c) shows the quantum correlation degradation in the collective phase-shift noise environment. It can be seen that the upper bound of rotating angles to destroy the quantum correlation property is 3.5° for the collective phase-shift noisy environment. Comparing the experimental results in the presences of different noisy channels, we can see that the quantum correlation is more robust under the phase-shift noisy environment than the bit-flip noises environment.

There are several open problems that require further investigation. First of all, it is natural to look for an extension of our result to higher-level systems. Moreover, it is interesting to study the robustness of information difference in multi-qubit or multi-qudit systems using the chained information Bell inequality. Finally, the information capacity of an entangled qubit pair in quantum information does not depend on the amount of entanglement only. It is very meaningful to investigate the robustness of the capacities in quantum information process using the information entropic criteria.

In summary, we have performed the first experimental demonstration of information-theoretic spin-1/2 inequalities using the high-fidelity entangled state. The concept that negative virtual information can be carried by entangled particles provides interesting insight into the information flow in quantum communication processes such as teleportation and superdense coding^33^. Furthermore, we first give the experimental rules of quantum correlation variation using the information difference of entropic criteria under bit-flip and phase-shift noises environment. The results improve our understanding of decoherence and will provide new strategies to control it. Our analysis offers new insights into the dynamics of entanglement in open systems.

## Methods

### Generation and optimization of the high-fidelity two-qubit entanglement state

We used the UV pulses of a frequency-doubled modelocked Ti:sapphire laser (pulse length 100 fs) to pump a 2 mm thick BBO crystal at a wavelength of 390 nm and a repetition rate of 80 MHz with an average power of 40 mW. The pump beam is focused to a waist of 100 um inside the crystal. Next, the photons were sent through the quantum channel, where the noisy environment was simulated by a combination of birefringent QWP and HWP in each arm. The HWP is switched angles *θ* and the QWPs are set at 0° with respect to the vertical direction. Then, the degenerate down-conversion emission passed through narrowband interference filters (3 nm) to exactly define the spatial and spectral emission modes. The polarization analysis is performed using further wave plates and polarizing beam splitters. Finally, the photons are coupled into single mode optical fibers and detected by silicon avalanche single photon detectors (D1 and D2). In order to improve the fidelity of entangled state, we have taken some methods: first, through many times experimental attempt, we get the optimal laser power, which can guarantee enough coincidence counting and visibility. Second, by inserting and fine tuning the QWP + HWP + QWP combination we can effectively improve the fidelity of entangled state. Finally, we get rid of the effect of dark count of measurement data.

## Additional Information

**How to cite this article**: Cao, L.-Z. *et al.* Experimental investigation of the information entropic Bell inequality. *Sci. Rep.* **6**, 23758; doi: 10.1038/srep23758 (2016).
